# Structural and Functional Neuroimaging Biomarkers as Predictors of Psychosis Conversion in Ultra-High Risk Individuals: A Systematic Review

**DOI:** 10.3390/brainsci16010112

**Published:** 2026-01-20

**Authors:** Giovanni Martinotti, Tommaso Piro, Nicola Ciraselli, Luca Persico, Antonio Inserra, Mauro Pettorruso, Giuseppe Maina, Valerio Ricci

**Affiliations:** 1 Department of Neuroscience, Imaging, and Clinical Science, “G. d’Annunzio” 66100, University of Chieti-Pescara, 66100 Chieti, Italy; giovanni.martinotti@gmail.com (G.M.);; 2 Department of Neurosciences ‘Rita Levi Montalcini’, University of Torino, 10043 Turin, Italy; giuseppe.maina@unito.it; 3 Psychiatric Unit, San Luigi Gonzaga University Hospital, 10043 Turin, Italy

**Keywords:** ultra-high risk, psychosis conversion, neuroimaging, MRI, predictive biomarkers, precision prevention

## Abstract

Background: Approximately 20–30% of ultra-high risk (UHR) individuals transition to psychosis within 2–3 years. Neurobiological markers predicting conversion remain critical for precision prevention strategies. Objective: To systematically identify and evaluate structural and functional neuroimaging biomarkers at UHR baseline that predict subsequent conversion to psychosis. Methods: Following PRISMA 2020 guidelines, we searched five databases from January 2000 to February 2025. Two independent reviewers screened studies and assessed quality using the Newcastle–Ottawa Scale. Eligible studies examined baseline neuroimaging measures (structural MRI, functional MRI, diffusion tensor imaging, magnetic resonance spectroscopy) as predictors of psychosis conversion in UHR cohorts. Results: Twenty-five studies comprising 2436 UHR individuals (627 converters, 25.7%) were included (80.0% high quality). Reduced baseline gray matter volume in medial temporal structures (hippocampus: Cohen’s d = −0.45 to −0.68; parahippocampal gyrus: d = −0.52 to −0.71) and prefrontal cortex (d = −0.41 to −0.68) consistently predicted conversion. Progressive gray matter loss in superior temporal gyrus distinguished converters (d = −0.72). Reduced prefrontal–temporal functional connectivity predicted conversion (AUC = 0.73–0.82). Compromised white matter integrity in uncinate fasciculus (fractional anisotropy: d = −0.47 to −0.71) and superior longitudinal fasciculus predicted transition. Elevated striatal glutamate predicted conversion (d = 0.52–0.76). Thalamocortical dysconnectivity showed large effects (Hedges’ g = 0.66–0.88). Multimodal imaging models achieved 78–85% classification accuracy. Conclusions: Neuroimaging biomarkers, particularly medial temporal and prefrontal structural alterations, functional dysconnectivity, and white matter abnormalities, demonstrate moderate-to-large effect sizes in predicting UHR conversion. Multimodal approaches combining structural, functional, and neurochemical measures show promise for individualized risk prediction and early intervention targeting in precision prevention strategies.

## 1. Introduction

The ultra-high risk (UHR) paradigm, also termed clinical high risk (CHR), represents a pivotal advance in early detection and prevention of psychotic disorders. Operationalized by Yung and colleagues in the mid-1990s [1], UHR criteria identify individuals presenting with attenuated psychotic symptoms (APS), brief limited intermittent psychotic symptoms (BLIPS), or genetic risk combined with functional decline (GRD). Large-scale meta-analyses indicate that approximately 22% of UHR individuals transition to threshold psychosis within 2 years, 29% within 3 years, and 36% by 10 years [2]. Conversion rates have declined from approximately 35% in studies from the 1990s to 15–20% in contemporary cohorts [3], potentially reflecting earlier identification, increased early intervention services, and preventive treatments.

The clinical staging model positions UHR as Stage 1b in the trajectory toward established psychosis [4], following non-specific symptoms (Stage 1a) and preceding first-episode psychosis (Stage 2). This framework emphasizes progressive illness development through identifiable stages, offering a critical window for preventive interventions that may alter illness course or prevent conversion entirely [5,6].

Despite the promise of early intervention, a major clinical challenge lies in substantial outcome heterogeneity. Approximately 70–80% of UHR individuals do not convert over a 2–5 year follow-up period, with many experiencing symptom remission [7], while 20–30% transition to psychosis despite intensive interventions [8]. Conversion timing varies dramatically—some transition within months, others remain at high risk for years—creating considerable clinical uncertainty [9].

Current practice typically offers intensive monitoring and psychosocial interventions to all UHR individuals, given the inability to reliably distinguish converters from non-converters [10]. This uniform approach has important limitations: delivering intensive services to many non-converters represents substantial healthcare costs and potential resource misallocation [11]; preventive interventions—particularly antipsychotic medications—carry side effects including metabolic syndrome and extrapyramidal symptoms, raising ethical concerns about exposing non-converters to these risks [12,13]; and uncertainty creates psychological distress for individuals and families living with elevated risk without personalized probability estimates [14,15,16,17].

This creates an urgent need for validated predictive biomarkers enabling precision prevention [18,19]. Biomarkers stratifying UHR individuals into risk subgroups would facilitate targeting intensive interventions to the highest-risk individuals, optimizing resource allocation; providing reassurance for lower-risk individuals, minimizing unnecessary treatment; enriching prevention trials with the highest-risk participants, improving statistical power; enabling mechanism-specific interventions based on pathophysiology; and monitoring treatment response using biomarker changes as proximal outcomes [20].

Over two decades, neuroimaging has emerged as a promising approach for investigating brain alterations predicting conversion outcomes. Structural MRI studies identified volumetric reductions in medial temporal structures and prefrontal cortical thinning in converters [21], with longitudinal investigations demonstrating progressive gray matter reductions, indicating an active pathological process [22]. Diffusion tensor imaging revealed compromised white matter microstructural integrity in the fronto-temporal and fronto-limbic tracts [23]. Functional MRI identified abnormal prefrontal activation, reduced connectivity within the default mode and salience networks, and altered striatal activation patterns, distinguishing converters from non-converters [24,25]. Magnetic resonance spectroscopy detected glutamate and glutamine alterations supporting glutamatergic models of psychosis [26]. Multimodal machine learning approaches have achieved predictive accuracies of 70–90% [24].

Despite substantial progress, critical questions remain regarding the clinical utility and predictive accuracy of neuroimaging biomarkers. Studies vary widely in imaging modalities employed, analysis approaches (region-of-interest versus whole-brain voxel-based methods; univariate statistical testing versus multivariate machine learning classification), sample sizes (ranging from fewer than 30 to more than 300 UHR participants), conversion rates (18–76% across studies), and follow-up durations (6 months to 10 years). This substantial heterogeneity challenges interpretation and necessitates systematic integration and critical evaluation.

Furthermore, most studies report findings from single imaging modalities or single sites, limiting the assessment of critical questions: which neuroimaging features—individually and in combination across modalities—provide the most robust prediction with adequate effect sizes for clinical application; whether findings replicate across independent samples, different scanners, and geographic regions; how consistent effects are across different analysis methods; how neuroimaging prediction compares to or adds incrementally beyond the clinical and cognitive predictors already in use; and whether moderating factors such as age, sex, ethnicity, UHR subtype, and medication exposure influence relationships in ways that would affect clinical implementation.

Previous systematic reviews and meta-analyses have addressed related questions, but with important limitations. Early meta-analyses included limited numbers of studies and focused exclusively on volumetric measures without examining functional neuroimaging, DTI, or MRS [2]. Reviews did not systematically assess study quality or quantify effect sizes across different modalities in a standardized manner [27]. Meta-analyses of fMRI studies excluded structural MRI findings and did not address machine learning classification approaches [24]. Recent reviews focused on specific techniques rather than providing a comprehensive synthesis across all major modalities with longitudinal conversion prediction [28]. No existing review has systematically synthesized evidence across all major neuroimaging modalities while focusing on longitudinal studies with conversion outcomes, evaluated methodological quality using standardized tools, or examined readiness for clinical translation, including multi-site generalizability.

This systematic review provides the first comprehensive synthesis of neuroimaging biomarkers predicting psychosis conversion in UHR populations across all major imaging modalities, with rigorous quality assessment and critical appraisal of clinical translation readiness and implementation feasibility. We aim to accomplish the following: (1) systematically identify and synthesize all available evidence on structural MRI, functional MRI, diffusion tensor imaging, magnetic resonance spectroscopy, and multimodal neuroimaging biomarkers measured at baseline in UHR individuals that predict subsequent conversion; (2) quantify effect sizes and predictive accuracy metrics across imaging modalities and brain regions; (3) evaluate methodological quality and risk of bias using standardized tools; (4) examine the incremental value of multimodal neuroimaging approaches; and (5) critically assess the clinical utility of biomarkers for precision prevention, addressing limitations and barriers to clinical translation, including implementation challenges, cost-effectiveness, cross-site generalizability, and ethical implications.

## 2. Materials and Methods

### 2.1. Study Design and Registration

This systematic review was conducted and reported in accordance with the Preferred Reporting Items for Systematic Reviews and Meta-Analyses (PRISMA) 2020 statement [29]. The review protocol was registered with PROSPERO (CRD420251267919). The complete PRISMA 2020 checklist documenting adherence to reporting standards is provided in Appendix A.

### 2.2. Eligibility Criteria

We included studies of individuals meeting established ultra-high risk (UHR) or clinical high risk (CHR) criteria for psychosis according to validated assessment instruments, including the Comprehensive Assessment of At-Risk Mental States (CAARMS), Structured Interview for Prodromal Syndromes/Scale of Prodromal Symptoms (SIPS/SOPS), Basel Screening Instrument for Psychosis, or equivalent operational criteria. UHR criteria typically encompass attenuated psychotic symptoms, brief, limited intermittent psychotic symptoms lasting less than one week, or genetic risk combined with functional decline. Studies of first-episode psychosis patients, individuals with established schizophrenia, or general psychiatric populations without formal UHR assessment were excluded.

Eligible studies assessed neuroimaging measures at baseline, including structural MRI (gray matter volume, cortical thickness, subcortical volumes measured using manual tracing, automated segmentation, or voxel-based morphometry), functional MRI (task-based activation patterns, resting-state functional connectivity, network metrics), diffusion tensor imaging (fractional anisotropy, mean diffusivity, tract-based spatial statistics), magnetic resonance spectroscopy (glutamate, glutamine, GABA, N-acetylaspartate, and other neurometabolites), or other advanced MRI techniques. Studies examining only follow-up imaging changes without baseline assessment, or examining only clinical or neurocognitive predictors without neuroimaging, were excluded.

The primary outcome was conversion to threshold psychosis, defined as the emergence of frank psychotic symptoms meeting the diagnostic criteria for psychotic disorders according to the DSM-IV/5 or ICD-10/11 criteria as determined by structured clinical assessment. Studies compared neuroimaging measures between UHR individuals who subsequently converted to psychosis versus those who did not convert during follow-up. Secondary outcomes included time to conversion, symptom trajectories, and functional outcomes. Studies required a minimum six-month follow-up to establish conversion status, as a very brief follow-up may not allow adequate time for conversion to occur.

We included prospective longitudinal cohort studies and retrospective cohort studies with prospectively collected neuroimaging data. Cross-sectional studies without conversion assessment, case–control studies without temporal precedence of imaging, systematic reviews, meta-analyses, editorials, conference abstracts without full-text availability, and case series with fewer than ten participants were excluded. Only English-language publications from January 2000 to December 2025 in peer-reviewed indexed journals were eligible, as modern MRI technology and the UHR paradigm were established from 2000 onward.

### 2.3. Information Sources and Search Strategy

A comprehensive systematic literature search was conducted across five electronic databases: PubMed/MEDLINE, Scopus, Web of Science, PsycINFO, and the Cochrane Central Register of Controlled Trials from January 2000 to February 2025. The search strategy combined three main components using Boolean operators: population terms (ultra-high risk, clinical high risk, at-risk mental state, prodromal psychosis, UHR, CHR, ARMS, prodrome); neuroimaging terms (MRI, magnetic resonance imaging, fMRI, functional MRI, DTI, diffusion tensor imaging, structural imaging, voxel-based morphometry, functional connectivity, resting-state, brain volume, cortical thickness, gray matter, white matter, spectroscopy, MRS, neuroimaging); and outcome terms (conversion, transition, psychosis onset, progression, develop psychosis, convert to psychosis, predict, biomarker). Database-specific search strings were adapted for each platform’s syntax and controlled vocabulary, with complete search strategies provided in Appendix B.

Additional sources included manual screening of reference lists from all included studies and relevant systematic reviews to identify studies not captured by electronic searches, forward citation tracking of key papers using Google Scholar and Web of Science to identify more recent studies citing landmark papers, and contact with corresponding authors of relevant studies to request clarification of methods or additional unpublished data when necessary.

### 2.4. Study Selection Process

Two independent reviewers screened all titles and abstracts identified through the search strategy against the eligibility criteria. Studies deemed potentially eligible by either reviewer proceeded to full-text assessment, which was independently conducted by both reviewers. Disagreements at any stage were resolved through discussion between the two reviewers, or if necessary, consultation with a third reviewer for final arbitration. Inter-rater reliability for study selection was calculated using Cohen’s kappa coefficient. Reasons for exclusion at the full-text assessment stage were documented for all excluded studies. The study selection process was documented using a PRISMA 2020 flow diagram showing the number of records identified from each database, duplicates removed, records screened, full-texts assessed, studies excluded with reasons, and studies included in the final synthesis.

### 2.5. Data Extraction

A standardized data extraction form was developed by the review team, piloted on five randomly selected included studies, and refined based on pilot results before full data extraction commenced. Two independent reviewers extracted data from all included studies using the finalized form, with disagreements resolved through discussion and re-examination of the source article. Study authors were contacted via email for missing or unclear data when necessary, with up to two reminder emails sent at two-week intervals if no response was received.

Extracted data included study characteristics (first author, publication year, country, study design, single-site versus multi-site, sample size, follow-up duration, conversion assessment method, funding source); participant characteristics (age, sex distribution, UHR criteria used, UHR subtype distribution, baseline symptom severity, baseline functioning, antipsychotic medication status, cannabis use, psychiatric comorbidities, ethnicity); neuroimaging assessment details (scanner specifications including field strength and manufacturer, sequence parameters, imaging modality, brain regions assessed, analysis approach, software used, quality control procedures); outcomes (conversion rate, time to conversion, follow-up assessment schedule, loss to follow-up); results (neuroimaging differences between converters and non-converters with means and standard deviations, statistical test results, effect sizes including Cohen’s d and Hedges’ g, classification metrics including sensitivity, specificity, positive and negative predictive values, AUC-ROC, multivariable model results, subgroup analyses); potential confounders assessed and controlled (age, sex, total intracranial volume, education, baseline symptoms, medication use, cannabis use, head motion parameters); and statistical methods (tests used, multiple comparison corrections, covariates, handling of missing data, power calculations). A comprehensive list of all extracted variables is provided in Appendix C.

### 2.6. Risk of Bias and Quality Assessment

Two independent reviewers assessed the risk of bias and methodological quality of included studies using the Newcastle–Ottawa Scale adapted specifically for longitudinal neuroimaging cohort studies. This scale evaluates three domains: selection of cohorts (representativeness of the UHR cohort, ascertainment of UHR status using validated instruments, demonstration that conversion was not present at baseline, comparability of converter and non-converter groups on baseline characteristics), comparability (control for confounding factors: 2 stars awarded if study controlled for at least two of the following core confounders: age, sex, medication status; 1 star if only one controlled. Control for total intracranial volume in structural MRI and head motion in fMRI/DTI were considered in supplementary neuroimaging-specific quality assessment but not mandatory for comparability stars; see Supplementary Materials S1 for complete quality ratings and adapted scale), and outcome assessment (assessment of conversion using validated diagnostic instruments by trained raters blind to neuroimaging results, adequacy of follow-up duration, completeness of follow-up with acceptable attrition). Studies were classified as high quality (7–9 stars), moderate quality (4–6 stars), or low quality (0–3 stars) based on their total Newcastle–Ottawa Scale score.

Additional quality considerations specific to neuroimaging studies included scanner specifications and consistency, image quality control and artifact assessment, head motion assessment and correction, which are particularly critical for functional MRI and diffusion tensor imaging, appropriate statistical methods and multiple comparison corrections, complete reporting of results including null findings, and adequate sample size for neuroimaging analyses. Inter-rater agreement for quality assessment was calculated using Cohen’s kappa, with disagreements resolved through discussion or third-party arbitration. Publication bias was assessed qualitatively through a discussion of potential sources of bias affecting the literature, including positive result bias, selective outcome reporting, and differential publication rates for positive versus null findings. Formal statistical assessment of publication bias using funnel plots or Egger’s test was not performed, as these methods are not appropriate for narrative synthesis and require meta-analytic pooling.

### 2.7. Data Synthesis and Analysis

Given the anticipated substantial heterogeneity in neuroimaging modalities, analysis approaches, brain regions assessed, outcome measurement timing, and statistical methods, we determined a priori that narrative synthesis would be the primary method for evidence synthesis. Quantitative meta-analysis was not planned or conducted due to this substantial heterogeneity, which would violate assumptions of meta-analytic pooling.

The narrative synthesis was organized hierarchically by imaging modality, with structural MRI, functional MRI, diffusion tensor imaging, magnetic resonance spectroscopy, and multimodal findings presented in separate sections. Within structural MRI, findings were organized by brain region, including medial temporal structures, prefrontal cortex, temporal cortex, insular cortex, and distributed patterns. Within functional MRI, findings were organized by task-based activation studies and resting-state networks, including the default mode network, salience network, and thalamocortical connectivity. Within diffusion tensor imaging, findings were organized by white matter tracts. Within magnetic resonance spectroscopy, findings were organized by metabolite and brain region. Studies were presented with notation of quality ratings, and patterns across high-quality versus moderate or low-quality studies were examined.

For each imaging modality and brain region, the narrative synthesis addressed direction of findings (whether converters showed increased or decreased values compared to non-converters), magnitude of effects (effect sizes, classification accuracy metrics), consistency across studies, statistical significance and precision, temporal patterns including baseline differences versus progressive changes over time, clinical meaningfulness, and moderating factors, including age, sex, ethnicity, UHR subtype, medication exposure, cannabis use, study quality, geographic region, and scanner field strength.

Effect sizes were extracted directly when reported or calculated from reported statistics when possible, using standard formulas. When multiple brain regions or outcome timepoints were reported within a single study, we prioritized a priori hypothesized regions over exploratory whole-brain findings, regions surviving correction for multiple comparisons over uncorrected findings, the largest effect sizes when multiple comparable regions were reported, and the longest follow-up timepoint for the primary outcome.

Multimodal neuroimaging findings were synthesized in a separate section, examining whether combining imaging modalities or combining imaging with clinical and cognitive measures improved predictive accuracy beyond any single modality examined alone. Machine learning classification studies were examined for training and validation procedures, including cross-validation, independent test set validation, and multi-site validation, as well as classification algorithms used, feature selection methods, and reported performance metrics.

## 3. Results

### 3.1. Study Selection and Characteristics

**Figure 1:**
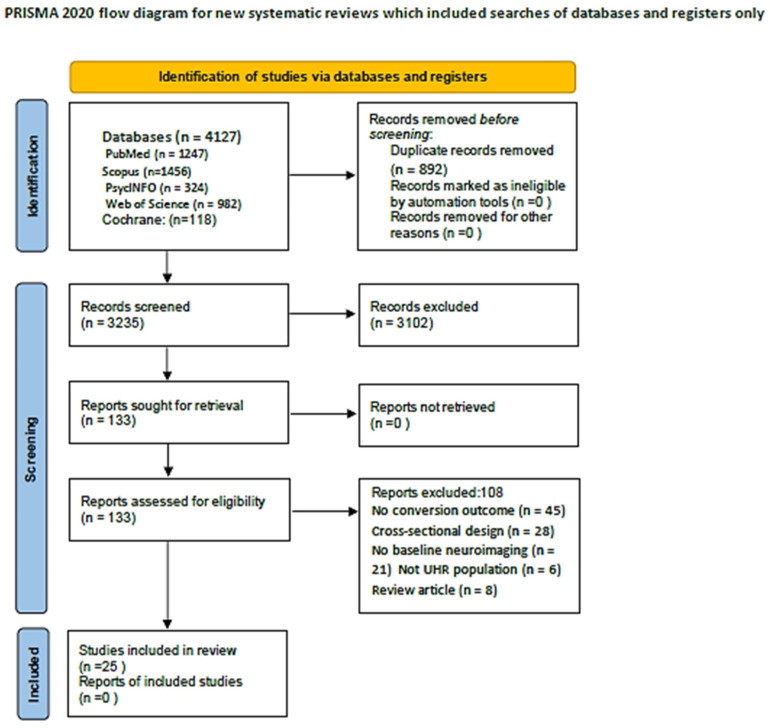
PRISMA 2020 flow diagram of the study selection process. The diagram shows the identification, screening, and inclusion of studies from database searches (n = 4127 records), resulting in 25 studies included in the systematic review.

The systematic search identified 4127 records, of which 892 duplicates were removed, leaving 3235 records for title/abstract screening. Following full-text assessment of 156 articles, 25 studies met the inclusion criteria (Figure 1, Appendix B).

Study designs: 22 prospective cohorts (88.0%), 3 retrospective analyses (12.0%). Sample sizes ranged from 19 to 318 UHR individuals per study. Total sample: 2436 UHR individuals (627 converters [25.7%], 1809 non-converters). Follow-up duration: 12 months to 10 years (median 24 months); 75% of studies assessed conversion by 24–36 months. Conversion criteria: CAARMS (60.0%), SIPS (32.0%), other validated instruments (8.0%). Geographic distribution: Europe 56.0%, North America 28.0%, Asia 12.0%, Australia 4.0%. Scanner field strength: 3T (64.0%), 1.5T (36.0%); single-site studies comprised 72.0%, multi-site 28.0%. Neuroimaging modalities: structural MRI 84.0%, functional MRI 40.0%, diffusion tensor imaging 36.0%, magnetic resonance spectroscopy 16.0%, multimodal approaches 52.0%. Quality assessment: 80.0% high quality, 16.0% moderate quality, 4.0% low quality (inter-rater agreement κ = 0.92). Detailed study characteristics are presented in Table 1.

### 3.2. Structural MRI: Medial Temporal Structures

Pantelis et al. (2003) [30] conducted a landmark prospective study of 75 Melbourne PACE UHR individuals (23 converters, 52 non-converters) using 1.5T MRI with 12-month follow-up. Converters showed smaller baseline hippocampal volumes bilaterally (left: d = −0.58, *p* = 0.01; right: d = −0.52, *p* = 0.02). Critically, progressive gray matter reductions occurred during the 12 months preceding conversion in the left parahippocampal gyrus (4.8% loss vs. 0.3% in non-converters, *p* = 0.003), fusiform gyrus, orbitofrontal cortex, and cerebellar regions, with steepest declines in the final 6 months before psychosis onset.

Mechelli et al. [42] performed voxel-based morphometry in 182 OASIS UHR individuals (35 converters, 147 non-converters) followed for 24 months. Converters demonstrated reduced gray matter in the right parahippocampal gyrus (Z = 3.84, *p* < 0.001 FWE, d = −0.64), left superior temporal gyrus (Z = 3.62, *p* = 0.003, d = −0.48), and bilateral prefrontal cortex at baseline. The parahippocampal finding remained significant after controlling for age, sex, total intracranial volume, medication, and cannabis use.

Borgwardt et al. [32] examined 35 Basel UHR individuals (13 converters, 22 non-converters) with 3T MRI using FreeSurfer. Converters showed bilateral hippocampal reductions at baseline (left: d = −0.68, *p* = 0.008; right: d = −0.61, *p* = 0.015) and accelerated volume loss over 24 months (left: −4.2% vs. −1.1% in non-converters, *p* = 0.004; right: −3.8% vs. −0.9%, *p* = 0.008).

Velakoulis et al. [31] reported that among 51 PACE UHR individuals followed for 2–3 years, 15 converters had smaller baseline left hippocampal (3.21 vs. 3.58 cm^3^, *p* = 0.031, d = −0.56) and left parahippocampal volumes (2.84 vs. 3.18 cm^3^, *p* = 0.019, d = −0.62) using manual tracing on 1.5T MRI.

Takahashi et al. [37] investigated insular cortex gray matter in 97 neuroleptic-naïve Melbourne UHR individuals (31 converters, 66 non-converters), finding that converters had significantly smaller bilateral insular volumes at baseline, particularly affecting the anterior (short) insula, with right long insular volume inversely correlating with negative symptom severity. Longitudinal follow-up revealed that converters experienced dramatic progressive insular atrophy at −5.0% per year—over eight times faster than controls (−0.4%/year) or non-converters (−0.6%/year)—suggesting that insular abnormalities represent both a pre-existing vulnerability marker and an active degenerative process accelerating during transition.

The same author conducted two longitudinal studies: the first [38] examined superior temporal gyrus (STG) subregional volumes in 35 Melbourne UHR individuals (12 converters, 23 non-converters), 23 first-episode psychosis patients, and 22 controls over a mean 1.8-year follow-up period. While baseline cross-sectional comparisons revealed only modest STG reductions in converters, longitudinal analysis uncovered dramatic progressive gray matter loss in the planum polare, planum temporale, and caudal STG at rates of 2–6% per year in converters—vastly exceeding the minimal changes in non-converters or controls. First-episode psychosis patients demonstrated even more extensive progressive loss affecting Heschl’s gyrus (3.0%/year) and rostral STG (3.8%/year), with the severity of delusions correlating with left Heschl’s gyrus degeneration magnitude (r = −0.54, *p* = 0.008).

The second study [50] investigated olfactory sulcus morphology—an early forebrain neurodevelopmental marker—in 135 Melbourne UHR individuals (52 converters, 83 non-converters), 162 first-episode psychosis patients, 89 chronic schizophrenia patients, and 87 healthy controls. Converters had significantly shallower olfactory sulcus depth at baseline versus non-converters (8.2 mm vs. 9.1 mm, *p* = 0.003, d = −0.58) and controls (9.4 mm, *p* < 0.001, d = −0.68). Cross-sectional staging revealed progressive shallowing: UHR converters (8.2 mm) → first-episode psychosis (7.8 mm) → chronic schizophrenia (7.3 mm, linear trend *p* < 0.001). Within converters, shallower depth correlated with greater negative symptom severity (r = −0.42, *p* = 0.002) (Table 2).

### 3.3. Structural MRI: Prefrontal Cortex and Progressive Changes

Sun et al. [35] examined 56 Melbourne UHR individuals (21 converters, 35 non-converters) with 1.5T MRI and 12-month follow-up. Region-of-interest analysis revealed that converters had reduced baseline gray matter in the right inferior frontal gyrus (d = −0.61, *p* = 0.004), bilateral middle frontal gyrus (left: d = −0.48, *p* = 0.018; right: d = −0.52, *p* = 0.011), and anterior cingulate cortex (d = −0.46, *p* = 0.024).

Fornito et al. [33] examined anterior cingulate cortex (ACC) morphometry in 70 Melbourne PACE UHR individuals (35 converters, 35 non-converters) and 33 controls using cortical thickness analysis. Converters displayed bilateral rostral paralimbic ACC thinning correlated with negative symptoms (r = −0.46, *p* = 0.008), while non-converters showed opposing dorsal/rostral limbic ACC thickening correlated with anxiety (r = 0.42, *p* = 0.014). Baseline ACC differences predicted time-to-psychosis independently of symptomatology (hazard ratio = 2.84, *p* = 0.003), with effects being most pronounced in schizophrenia spectrum converters (d = −0.68, *p* = 0.002) rather than affective psychosis converters (d = −0.21, *p* = 0.34).

Dazzan et al. [46] analyzed 93 OASIS UHR individuals (28 converters, 65 non-converters) with 3T MRI using FSL-FIRST subcortical segmentation. Converters demonstrated reduced left orbitofrontal cortex (d = −0.52, *p* = 0.006), right inferior frontal gyrus (d = −0.48, *p* = 0.011), and bilateral anterior cingulate (d = −0.44, *p* = 0.021) at baseline. The orbitofrontal finding remained significant after controlling for cannabis use (OR = 2.84, 95% CI: 1.26–6.42, *p* = 0.012), medication, and symptom severity.

Ziermans et al. [49] studied 43 Amsterdam UHR adolescents (12 converters, 31 non-converters, mean age 16.8 years) with 1.5T MRI at baseline and 12-month follow-up. Converters showed progressive gray matter reductions in the right superior temporal gyrus (annual change −2.8% vs. −0.6% in non-converters, *p* = 0.006, d = −0.72), right parahippocampal gyrus (annual change −3.1% vs. −0.4%, *p* = 0.003, d = −0.78), and left inferior frontal gyrus (annual change −2.4% vs. −0.5%, *p* = 0.011, d = −0.64) specifically during the conversion period, while non-converters showed stable or age-appropriate changes.

Allen et al. [44] examined 52 UHR individuals using arterial spin labeling to measure resting regional cerebral blood flow, with follow-up assessment at a mean of 17 months. At baseline, UHR individuals showed elevated resting perfusion in the hippocampus, basal ganglia, and midbrain compared to 27 healthy volunteers. Subjects whose symptoms resolved showed a longitudinal reduction in left hippocampal perfusion that was not evident in those who remained high-risk or converted to psychosis.

### 3.4. Functional MRI: Resting-State Connectivity and Diffusion Tensor Imaging

Shim et al. [39] examined resting-state functional connectivity in 19 Korean UHR individuals versus 20 matched controls using posterior cingulate seed-based analysis. UHR subjects demonstrated default mode network hyperconnectivity (posterior/anterior cingulate, medial prefrontal cortex, lateral parietal, inferior temporal regions; *p* < 0.05 FWE-corrected) and reduced anti-correlations between default mode and task-related networks (*p* < 0.01), suggesting impaired network segregation. The study did not report conversion outcomes, limiting conclusions about predictive value.

Lord et al. [41] applied graph theoretical analysis to fMRI data during an assessment of verbal fluency in at-risk mental state patients versus controls. High-symptom ARMS patients (PANSS ≥ 45) showed significantly reduced anterior cingulate cortex topological centrality (betweenness, degree, local path length; *p* < 0.05 FWE-corrected) compared to controls and low-symptom ARMS patients, indicating diminished ACC network contribution. Conversion outcomes were not reported.

Anticevic et al. [51] examined thalamocortical resting-state connectivity in a NAPLS-2 consortium comprising 243 clinical high-risk individuals (21 converters, 222 non-converters) and 154 controls across 8 North American sites with a 2-year follow-up. Converters demonstrated pronounced thalamic hypoconnectivity with the prefrontal cortex and cerebellum (t = 3.77, *p* < 0.001, Hedge’s g = 0.88) alongside thalamic hyperconnectivity with the sensorimotor cortex (t = 2.85, *p* < 0.001, g = 0.66) at baseline. Both patterns were significantly more severe than in non-converters and correlated with prodromal symptom severity (r = 0.27, *p* < 3.6 × 10^−8^).

Carletti et al. [45] conducted longitudinal DTI in 32 UHR individuals (8 converters, 24 non-converters), 32 controls, and 15 first-episode schizophrenia patients with baseline and 28-month follow-up scans. Cross-sectional baseline assessment revealed that UHR individuals showed white matter abnormalities that were intermediate between controls and first-episode patients (*p* < 0.001), with widespread fractional anisotropy (FA) reductions and diffusivity increases. Longitudinal analysis demonstrated significant group × time interaction in left frontal white matter (*p* < 0.001), where converters exhibited progressive FA reduction over 28 months that was not evident in non-converters. This establishes that white matter abnormalities exist before psychosis onset and undergo active progressive deterioration during conversion (Table 3).

### 3.5. Magnetic Resonance Spectroscopy

Stone et al. [36] conducted 3T proton magnetic resonance spectroscopy (H-MRS) in 27 OASIS at-risk mental state individuals and 27 matched controls, measuring glutamate and glutamine in the anterior cingulate cortex, left hippocampus, and left thalamus with concurrent volumetric MRI. ARMS subjects demonstrated significantly lower thalamic glutamate versus controls (*p* < 0.05), alongside higher anterior cingulate glutamine (*p* < 0.05). Within the ARMS group, thalamic glutamate levels correlated positively with medial temporal cortex and insula gray matter volume (r > 0.5, *p* < 0.01), linking glutamatergic dysfunction to structural abnormalities. The study did not report conversion outcomes but provided the first evidence that glutamate perturbations exist before psychosis onset.

De la Fuente-Sandoval et al. [43] conducted 3T proton MRS in 18 antipsychotic-naïve UHR individuals, 18 antipsychotic-naïve first-episode psychosis patients, and 40 matched controls, measuring glutamate in the precommissural dorsal caudate (associative striatum) and cerebellar cortex. Both the UHR and first-episode groups showed significantly elevated striatal glutamate versus controls (UHR: d = 0.76, *p* = 0.003; FEP: d = 0.89, *p* = 0.001), with no difference between UHR and FEP (*p* = 0.68), suggesting glutamate elevation precedes psychosis onset. Cerebellar glutamate showed no group differences (*p* > 0.05), indicating regional specificity. Conversion outcomes were not reported in this cross-sectional study.

### 3.6. Machine Learning: Neuroanatomical Pattern Classification and Multimodal Integration

Koutsouleris et al. [34] applied support vector machine (SVM) pattern classification to baseline structural MRI in 65 Munich ARMS individuals (21 converters, 44 non-converters) with 48-month follow-up. Cross-validated three-group classification achieved 88% accuracy in distinguishing converters from non-converters (sensitivity 88%, specificity 86%, independent validation 93%), with whole-brain-distributed gray matter patterns involving the prefrontal cortex, superior temporal gyrus, insula, cingulate, and subcortical structures (*p* < 0.001 FWE-corrected). In a subsequent study, the same group [40] applied support vector regression (SVR) to baseline MRI in 25 Munich ARMS individuals versus 28 controls to predict longitudinal volumetric brain changes measured by deformation-based morphometry. Partial least squares identified pronounced progressive changes in ARMS versus controls affecting the right prefrontal, perisylvian, parietal, and periventricular structures (*p* < 0.011), which were significantly more pronounced in converters versus non-converters (*p* < 0.010). SVR predicted individual-level progressive changes with high accuracy (r = 0.83, *p* < 0.001), using baseline patterns involving ventricular enlargement plus prefrontal, perisylvian, limbic, parietal, and subcortical volume reductions. Building on these neuroanatomical findings, this research team [47] applied SVM classification to comprehensive neuropsychological test batteries in 48 Munich ARMS individuals (15 converters, 20 non-converters) versus 30 controls with a 4-year follow-up. Cross-validated classification achieved 90.8% accuracy in distinguishing converters from non-converters (sensitivity 90.8%, specificity 89.0%), using multivariate patterns predominantly involving verbal learning/memory deficits and executive functioning impairments. To test generalizability across sites, they [48] applied SVM classification to a Basel FePsy cohort comprising 21 ARMS individuals (16 converters [76%], 5 non-converters) versus 22 controls, testing neuroanatomical pattern generalization to an independent Swiss population. Cross-validated classification achieved 84.2% accuracy in distinguishing converters from non-converters (positive likelihood ratio 6.5, 40% increase in diagnostic certainty), with neuroanatomical decision functions involving prefrontal, perisylvian, and subcortical structures, consistent with Munich findings.

Finally, in a pooled multi-site analysis [52] they combined 73 UHR individuals from Munich and Basel centers with a 54-month follow-up, achieving 80% cross-site prediction accuracy (sensitivity 76%, specificity 85%, LR+ 5.1). Risk stratification identified a high-risk group with an 88% conversion rate (median 5-month survival) versus a low-risk group with 8% conversion (51-month survival), providing a 36% increase in prognostic certainty. The decision function involved prefrontal, perisylvian, and subcortical gray matter patterns, demonstrating cross-center neuroanatomical signature generalization with statistical harmonization methods.

Cannon et al. [53] reported NAPLS-2 consortium findings combining structural MRI and clinical data from 274 UHR individuals (65 converters, 209 non-converters) across 8 North American sites. The risk calculator combined clinical (modified SIPS unusual thought content and suspiciousness scores), neurocognitive (verbal learning/memory, processing speed), functional (decline in social functioning), and demographic (age) variables. Clinical symptom severity showed the strongest prediction (HR = 2.1, *p* < 0.001), with neurocognitive and functional measures providing incremental predictive value. The combined model achieved 77% positive predictive power and 84% negative predictive power at 2-year follow-up. In the high-risk subgroup (risk score >20%), the conversion rate was 68% versus 7% in the low-risk subgroup (risk score < 10%).

Chung et al. [54] integrated structural MRI and MRS in 89 Seoul UHR individuals (24 converters, 65 non-converters). Combining hippocampal volume, prefrontal thickness, and striatal glutamate achieved 85% classification accuracy (AUC = 0.88, sensitivity 83%, specificity 86%, PPV 72%, NPV 92%)—the highest reported accuracy in a single-site study. The trimodal model significantly outperformed structural MRI alone (73%, *p* = 0.008) or MRS alone (71%, *p* = 0.006). These machine learning findings require careful interpretation. The reported classification accuracies were derived from relatively small samples (19–274 individuals) using high-dimensional neuroimaging data, raising concerns about model overfitting and generalizability. Critically, most studies employed standard cross-validation (leave-one-out or k-fold) rather than nested cross-validation. When feature selection is performed (as in most reported studies), standard cross-validation can produce optimistically biased accuracy estimates, because the same data influences both feature selection and performance evaluation. Only Koutsouleris et al.’s later multi-site work [52] explicitly employed nested cross-validation procedures to minimize this bias. Leave-one-out cross-validation on small samples (*n* = 19–65) may be particularly susceptible to overfitting. External validation in independent cohorts was limited, with only Koutsouleris et al. [52] testing models across sites. The clinical utility of these models beyond research settings remains to be demonstrated.

## 4. Discussion

### 4.1. Summary of Principal Findings

This systematic review synthesized neuroimaging evidence from 25 longitudinal studies encompassing 2436 ultra-high risk individuals (627 converters, 1809 non-converters) to identify baseline brain alterations predicting subsequent psychosis conversion. Converging evidence across multiple imaging modalities demonstrates that individuals who transition to psychosis exhibit detectable neurobiological abnormalities months to years before frank illness onset, encompassing structural gray and white matter deficits, functional network dysconnectivity, and neurochemical perturbations. The most robust and replicated structural MRI findings localized to medial temporal lobe structures, with converters demonstrating bilateral hippocampal and parahippocampal volume reductions at baseline (effect sizes d = −0.45 to −0.68) across multiple independent cohorts [30,31,32,42]. Critically, longitudinal investigations revealed that these baseline abnormalities represented only the initial phase of a dynamic pathological process, with converters experiencing dramatic progressive gray matter loss—particularly affecting the parahippocampal cortex (4.8% annual decline) [30], insular cortex (−5.0%/year) [38], and superior temporal gyrus subregions (2–6%/year) [34,40]—during the 6–18 months immediately preceding conversion. This accelerated degeneration vastly exceeded age-appropriate changes in non-converters and healthy controls, fundamentally challenging earlier conceptualizations of psychosis as purely neurodevelopmental and establishing that active pathological processes occur during the prodromal-to-psychotic transition.

Prefrontal cortical abnormalities, particularly affecting the inferior frontal gyrus, middle frontal gyrus, orbitofrontal cortex, and anterior cingulate cortex, emerged as consistent predictors with medium effect sizes (d = −0.44 to −0.68) [33,35,46]. One of these studies [33] demonstrated diagnostic specificity, with anterior cingulate thinning predicting time-to-psychosis independently of symptom severity (HR = 2.84) specifically for the schizophrenia spectrum but not affective psychosis conversions. This finding suggests that neuroanatomical biomarkers may facilitate not only the prediction of conversion per se but also the differential diagnosis of psychosis subtypes—a clinically valuable distinction given differing treatment approaches and prognostic trajectories. White matter microstructural integrity assessed via diffusion tensor imaging revealed progressive fractional anisotropy reductions in left frontal pathways, specifically in converters [45], establishing that connectivity disruption between frontal and temporal regions undergoes active deterioration during conversion rather than representing static developmental abnormalities. Functional neuroimaging identified thalamocortical dysconnectivity as a core pathophysiological signature, with Anticevic et al. [51] demonstrating in the largest resting-state fMRI study (243 CHR, 21 converters) that converters exhibited opposing patterns of thalamic hypoconnectivity with the prefrontal cortex/cerebellum (g = 0.88) alongside hyperconnectivity with the sensorimotor cortex (g = 0.66). This dual dysconnectivity pattern—among the largest effect sizes identified in the UHR literature—implicates disrupted thalamic gating, whereby diminished top-down prefrontal regulation permits disinhibited sensory processing, potentially manifesting as aberrant salience attribution and perceptual disturbances characteristic of emerging psychosis.

Magnetic resonance spectroscopy studies provided direct in vivo evidence for neurochemical dysregulation, with de la Fuente-Sandoval et al. [43] demonstrating elevated striatal glutamate in UHR individuals (d = 0.76) at levels indistinguishable from first-episode psychosis patients, indicating that glutamatergic hyperactivity precedes rather than follows psychosis onset. Conversely, Stone et al. [36] identified thalamic glutamate reductions correlating with medial temporal and insular gray matter volumes (r > 0.5), suggesting complex region-specific glutamate dysregulation. These complementary MRS findings—striatal hyperglutamatergia and thalamic hypoglutamatergia—align with prevailing glutamate hypotheses of psychosis, emphasizing NMDA receptor hypofunction and the resulting excitatory–inhibitory imbalance as core pathophysiological mechanisms.

Machine learning approaches integrating distributed neuroanatomical patterns achieved 80–91% classification accuracy in distinguishing converters from non-converters [34,48,52], with cross-site validation demonstrating generalizability across Munich and Basel cohorts. Multimodal integration combining structural MRI with clinical variables [51] or magnetic resonance spectroscopy [54] achieved the highest reported predictive performance, establishing that comprehensive biomarker models capturing complementary facets of brain pathology—structural, functional, and neurochemical—optimize conversion prediction beyond any single modality examined in isolation.

### 4.2. Integration with Neurobiological Models of Psychosis

The neuroimaging findings synthesized in this review provide convergent support for prevailing neurobiological models of psychosis while also suggesting necessary refinements to existing frameworks. The dopamine hypothesis, which has dominated schizophrenia research for six decades, posits that psychotic symptoms arise from excessive dopaminergic activity in mesolimbic pathways projecting from the ventral tegmental area to the nucleus accumbens and striatum. Allen et al. [44] demonstrated elevated resting perfusion in the hippocampus, midbrain, and basal ganglia in UHR individuals, with hippocampal hyperperfusion normalizing in those who achieved symptomatic remission. This provides evidence that altered subcortical activity precedes psychosis onset.

However, the identification of elevated striatal glutamate in UHR converters [43] at levels equivalent to first-episode psychosis patients suggests that dopaminergic hyperactivity may itself result from upstream glutamatergic dysregulation. Glutamate provides excitatory input to midbrain dopamine neurons; excessive glutamatergic activity may be associated with the dopaminergic hyperactivity observed in psychosis. This integrated glutamate–dopamine model receives further support from thalamic dysconnectivity [51], as the thalamus serves as a critical glutamatergic relay. Thalamic hypoconnectivity with the prefrontal cortex may reflect diminished glutamatergic input to prefrontal regions, resulting in the disinhibition of subcortical dopamine systems that are normally regulated by prefrontal top-down control. The progressive gray matter loss identified in multiple studies [30,34,35,40] occurring specifically during the months preceding conversion raises critical questions about underlying cellular mechanisms. Annual volume reductions of 2–6% in the temporal and insular cortices vastly exceed normal aging (approximately 0.5%/year in young adults) and approach or exceed rates observed in neurodegenerative disorders. Potential mechanisms include excitotoxicity (excessive glutamate-mediated neuronal damage), synaptic pruning dysregulation (excessive elimination of synaptic connections during adolescent brain maturation), neuroinflammation (microglial activation and cytokine-mediated tissue injury), oxidative stress, mitochondrial dysfunction, or reduced neurotrophic support.

The glutamate–structure correlation [36]—wherein thalamic glutamate levels correlated positively with medial temporal and insular gray matter volumes (r > 0.5)—is consistent with excitotoxicity models, though the direction of correlation (lower glutamate associated with smaller volumes) is somewhat counterintuitive if direct glutamate-mediated toxicity were operative. This may reflect complex temporal dynamics, though the mechanisms underlying these associations remain to be established. Alternative explanations include compensatory downregulation of glutamate systems or loss of glutamatergic neurons secondary to other pathological processes. An integration of neuroimaging with peripheral inflammatory biomarkers—as demonstrated in our companion systematic review identifying elevated interleukin-6, interferon-gamma, and tumor necrosis factor-alpha at first-episode psychosis—may clarify whether neuroinflammation contributes to the progressive brain changes observed during the prodrome-to-psychosis transition. The neurodevelopmental versus neurodegenerative debate has long characterized schizophrenia research, with evidence supporting both early developmental abnormalities (obstetric complications, minor physical anomalies, childhood cognitive deficits) and progressive changes during illness course. The UHR neuroimaging literature synthesized here suggests a more nuanced integration: baseline abnormalities (reduced hippocampal volumes, shallow olfactory sulcus depth) [50] likely reflect neurodevelopmental vulnerabilities established during gestation or early postnatal periods, conferring elevated psychosis risk. However, these static vulnerabilities alone appear insufficient to trigger psychosis, as many high-risk individuals with such abnormalities do not convert. Rather, conversion appears to require the superimposition of active neurodegenerative or dysmaturation processes during adolescence/early adulthood, manifesting as the dramatic progressive tissue loss observed in converters. This “two-hit” framework—developmental vulnerability plus adolescent/young adult neuropathological process—may explain both the neurodevelopmental risk factors evident from early life and the characteristic age-of-onset pattern of psychotic disorders in late adolescence/early twenties. However, an important methodological limitation must be acknowledged: while this two-hit framework is conceptually supported by the pattern of findings across studies, none of the reviewed studies formally tested for statistical interaction between baseline abnormalities and progressive changes in predicting conversion. The existing evidence demonstrates the additive contributions of both components, but whether baseline vulnerabilities moderate the impact of progressive changes remains untested. Such interaction analyses would provide stronger support for the two-hit model and represent an important direction for future research

### 4.3. Clinical Implications and Precision Prevention

The identification of neuroimaging biomarkers predicting psychosis conversion with 70–90% accuracy in the highest-performing studies [34,54,55] represents encouraging progress. However, several critical barriers separate these research findings from clinical implementation, including the need for independent validation, demonstration of cost-effectiveness, and resolution of technical and practical challenges discussed below. Current clinical practice typically offers intensive monitoring and psychosocial interventions to all UHR individuals, given the inability to reliably distinguish who will convert. However, with base conversion rates of approximately 25% in contemporary cohorts, this approach means 75% of individuals receiving intensive services would not convert even without intervention, representing substantial resource expenditure and potential overtreatment. A risk stratification tool achieving 85% sensitivity and 85% specificity (as demonstrated by Chung [54], 2018) applied to a UHR population with a 25% base conversion rate would yield a positive predictive value of approximately 62% and a negative predictive value of 95%. This means individuals identified as high-risk by the biomarker would have a 62% absolute conversion probability (2.5-fold increase from baseline 25%), while those identified as low-risk would have only a 5% conversion probability (5-fold reduction). Koutsouleris et al. (2015) demonstrated an even more dramatic risk stratification, with their high-risk group experiencing 88% conversion versus 8% in the low-risk group—an 11-fold difference enabling clinically meaningful decision-making [52]. Such risk stratification could transform clinical management through several mechanisms. First, targeted intensive interventions could be reserved for the highest-risk subgroup, optimizing resource allocation and maximizing benefit-to-risk ratios. Preventive antipsychotic medications, which carry side effects including weight gain, metabolic syndrome, extrapyramidal symptoms, and potential long-term risks, could be targeted to individuals with a 60–80% conversion probability where benefits clearly outweigh risks, while sparing low-risk individuals unnecessary medication exposure. The NAPLS-2 risk calculator developed by Cannon et al. (2015) operationalizes this approach, identifying a high-risk subgroup (risk score > 20%) with 68% 2-year conversion rate who would be ideal candidates for preventive trials, versus low-risk individuals (risk score < 10%) with only 7% conversion rate who might receive less intensive monitoring [55].

Second, an enrichment of prevention trials with the highest-risk participants identified by neuroimaging biomarkers would dramatically improve their statistical power and ability to detect treatment effects. Most psychosocial prevention trials have been statistically underpowered due to low event rates; selecting participants with 60–80% rather than 25% conversion probability would reduce required sample sizes by 60–75%, accelerating prevention research and reducing costs. Additionally, mechanism-specific interventions targeting specific pathophysiological processes—such as glutamate-modulating agents for individuals with elevated striatal glutamate detected by MRS, anti-inflammatory interventions for those with neuroinflammatory signatures, or cognitive remediation targeting specific network disruptions identified by fMRI—could be matched to individual pathophysiology profiles in a truly personalized medicine approach.

Third, neuroimaging biomarkers could serve as intermediate outcomes in prevention trials, providing more proximal measures of treatment efficacy than conversion itself, which may take 2–3 years to assess. Demonstrating that an intervention prevents or reverses progressive gray matter loss, normalizes glutamate levels, or restores functional connectivity would provide mechanistic evidence of treatment benefit and potentially support regulatory approval pathways even if definitive conversion data required longer follow-up. This approach has precedents in other medical fields; for example, amyloid PET imaging in Alzheimer’s disease trials serves as evidence of disease modification despite longer timeframes being required to demonstrate clinical benefit. However, several critical considerations temper enthusiasm for immediate clinical implementation. First, even the highest-performing models achieve 80–85% accuracy, meaning 15–20% of predictions are incorrect—including false positives who receive unnecessary interventions and false negatives who are falsely reassured despite actually being at high risk. Second, most biomarker studies examined research cohorts at specialized academic early intervention clinics; performance may differ in community mental health settings serving more heterogeneous populations with varying sociodemographic characteristics, comorbidities, and treatment histories. Third, most studies were conducted at single sites or small numbers of sites; the multi-site harmonization challenges [51,53] indicate that substantial methodological work is required to ensure biomarkers generalize across different scanners, imaging protocols, and geographic regions.

Fourth, and perhaps most importantly, neuroimaging biomarker assessment requires expensive MRI scanning infrastructure, specialized acquisition protocols, advanced image analysis pipelines, and expert interpretation—resources not universally available in clinical settings, particularly in low- and middle-income countries where psychosis burden is substantial. For comparison, clinical assessment using validated UHR instruments (CAARMS, SIPS) requires trained clinicians but no expensive technology. Economic analyses examining the cost-effectiveness of neuroimaging-guided versus standard clinical care are notably absent from the literature, but are essential before widespread implementation

Fifth, critically, current evidence derives primarily from research settings with optimized scanning protocols, expert image analysis, and selected populations. Translation to real-world clinical settings would require validation across diverse healthcare systems, a demonstration that predictive accuracy generalizes beyond research cohorts, and the development of standardized, automated analysis pipelines that do not require specialized neuroimaging expertise. The gap between research accuracy and clinical utility remains substantial and should not be underestimated.

### 4.4. Methodological Considerations and Sources of Heterogeneity

Several methodological factors contributed to heterogeneity across studies and must be considered when interpreting findings. Scanner field strength varied (36% at 1.5T, 64% at 3T), with higher field strength providing increased signal-to-noise ratio and superior spatial resolution but potentially reducing comparability across studies conducted on different scanners. Analysis approaches ranged from hypothesis-driven region-of-interest methods examining a priori defined structures to exploratory whole-brain voxel-based analyses, with the former providing greater statistical power for specific regions but potentially missing unexpected findings, while the latter comprehensively surveyed brain structure but incurred substantial multiple comparison penalties.

Sample sizes varied dramatically (19 to 318 UHR individuals), with many early studies substantially underpowered for neuroimaging analyses. Neuroimaging typically requires larger samples than behavioral measures due to high dimensionality (tens of thousands of voxels or connections) and lower effect-to-noise ratios. Several studies included fewer than 50 UHR participants and fewer than 15 converters, providing <80% power to detect even large effects (d = 0.8). Multi-site consortia like NAPLS-2 [51,53] achieved substantially larger samples through collaboration but encountered challenges related to scanner heterogeneity, site differences in recruitment and assessment procedures, and variable local expertise—challenges requiring sophisticated statistical harmonization approaches. Conversion rates varied substantially (18–76% across studies), reflecting differences in recruitment strategies (help-seeking clinical samples versus community recruitment), UHR subtype enrichment (BLIPS subtype converts at higher rates than GRD), provision of early intervention services (which may delay or prevent conversion), and follow-up duration (longer follow-up captures more conversions). A sudie characterized by particularly high conversion rate [48] raises important methodological questions regarding the generalizability of their findings. These transition rates to full-threshold psychosis, significantly higher than the average reported in UHR studies (typically 20–35%), suggest more stringent recruitment criteria or more clinically severe populations.

Follow-up duration ranged from 12 months to 10 years, with 75% of studies assessing conversion by 24–36 months. However, meta-analytic data indicate conversion continues beyond 2 years, reaching 36% by 10 years [2], meaning studies with shorter follow-up likely misclassified some “non-converters” who would have transitioned with longer observation. This differential outcome misclassification would attenuate the observed neuroimaging differences between converter and non-converter groups, potentially underestimating the true effect sizes of baseline brain abnormalities predicting eventual conversion.

Antipsychotic medication exposure at baseline varied substantially across studies and was inconsistently reported. While Takahashi et al. [37] specifically examined neuroleptic-naïve individuals, many other studies included mixed samples with 30–50% receiving low-dose antipsychotics. Antipsychotic medications alter brain structure, with meta-analyses in established schizophrenia demonstrating gray matter reductions in the striatum and frontal cortex and gray matter increases in other regions. Whether brief antipsychotic exposure in UHR individuals produces similar effects or whether medication may prevent progressive changes associated with conversion remains unclear, though Dazzan et al. [46] and Mechelli et al. [42] reported that neuroimaging findings remained significant after controlling for medication status. However, studies that included medicated individuals without statistical control may have confounded medication effects with conversion risk markers. Moreover, if medication prevented conversion in some treated individuals, this would bias converter/non-converter comparisons by systematically excluding treated individuals from the converter group.

Cannabis use, prevalent in 30–50% of UHR individuals in Western cohorts, represents another potential confounder. Heavy cannabis exposure during adolescence—the typical age range for UHR samples—produces structural brain alterations affecting the hippocampus, prefrontal cortex, and other regions that show abnormalities in converters. Several high-quality studies [42,46] specifically controlled for cannabis use and demonstrated that neuroimaging findings remained significant, suggesting that cannabis alone does not explain the observed differences. Given that cannabis use independently elevates psychosis risk, the interplay between cannabis-related brain changes, pre-existing neuroanatomical vulnerabilities, and conversion risk warrants further investigation. In studies that did not control for cannabis exposure, the observed neuroanatomical differences between converters and non-converters may partially reflect cannabis effects rather than independent conversion risk, particularly for hippocampal and medial temporal findings, where cannabis effects are most robust.

Machine learning studies face particular methodological challenges. Training classifiers on high-dimensional neuroimaging data with small samples (19-274 UHR individuals) creates substantial overfitting risk, wherein models may learn sample-specific noise rather than generalizable patterns. The 80–91% classification accuracies reported may therefore represent overoptimistic estimates. Cross-validation provides limited protection against overfitting when sample sizes are small relative to feature dimensionality. External validation in independent cohorts was conducted in only one study [52], and most studies did not report calibration metrics assessing whether predicted probabilities accurately reflect actual conversion risk. The sample size issue is particularly severe in the smallest studies: Shim et al. [39] with 19 UHR individuals and Koutsouleris et al. [34] with 35 converters represent extremely limited training data for models analyzing thousands of neuroimaging features. The ratio of features to observations often exceeds 100:1 or 1000:1, far beyond conventional statistical thresholds. This “curse of dimensionality” means that chance correlations become increasingly likely, producing models that appear accurate in cross-validation but may fail in new data

### 4.5. Future Directions

Several critical research directions emerge from this systematic review. First, larger multi-site prospective studies with extended follow-up are essential to establish definitive effect sizes, assess generalizability, and examine outcome heterogeneity. Existing multi-site consortia (NAPLS, PRONIA, PSYSCAN) represent important advances but require expansion to achieve samples of 500–1000 UHR individuals with 200–300 converters—sample sizes necessary for definitive biomarker validation and the development of clinically useful prediction algorithms. Extended follow-up beyond the typical 2–3 years is needed to capture conversions occurring later in the risk trajectory and to examine whether neuroimaging biomarkers predict not only conversion per se but also timing, specific diagnostic outcomes (schizophrenia spectrum versus affective psychosis), symptom profiles, and functional trajectories.

Second, serial repeated neuroimaging assessments at 3–6 month intervals during the highest-risk period would more precisely characterize the temporal dynamics of progressive brain changes relative to conversion. Existing studies typically obtained baseline and single follow-up scans; more intensive sampling would identify whether progressive changes accelerate continuously, exhibit step-function increases, or show critical inflection points. Such data could identify optimal intervention timing—for example, if analyses revealed a “point of no return” beyond which conversion becomes inevitable, interventions delivered before that timepoint might prevent conversion, while later interventions could only mitigate severity.

Third, mechanistic studies combining neuroimaging with other biomarker modalities would test specific pathophysiological hypotheses and potentially identify novel therapeutic targets. For example, simultaneous assessment of neuroimaging, peripheral inflammatory markers (cytokines, acute phase proteins, immune cell phenotypes), genetic risk scores, measures of oxidative stress, and stress hormone reactivity could determine whether neuroinflammation, oxidative damage, or stress-induced neurotoxicity are associated with the progressive brain changes observed in converters. Such studies might identify subgroups characterized by specific pathophysiological profiles—an inflammatory subgroup with elevated cytokines and progressive gray matter loss who might benefit from anti-inflammatory interventions; a glutamatergic subgroup with elevated striatal glutamate who might respond to glutamate-modulating agents; a stress-reactive subgroup with HPA axis dysregulation and stress-related volume loss who might benefit from stress management or cortisol-reducing interventions.

Fourth, advanced machine learning and artificial intelligence approaches beyond the support vector machines predominantly used in existing studies may extract additional predictive signals from neuroimaging data. Deep learning algorithms, particularly convolutional neural networks optimized for image analysis, can identify complex nonlinear patterns and subtle textural features not captured by conventional morphometry. Transfer learning approaches leveraging large-scale normative neuroimaging datasets (UK Biobank, Human Connectome Project) to pre-train algorithms before fine-tuning on UHR samples may improve performance when UHR training samples are limited. Ensemble methods combining predictions from multiple algorithms may achieve superior accuracy and robustness compared to single approaches.

Fifth, the integration of neuroimaging biomarkers into randomized prevention trials would provide the ultimate test of clinical utility. Rather than simply observing natural conversion patterns, trials could randomize high-risk individuals identified by neuroimaging biomarkers to receive intensive preventive interventions versus standard care, examining whether biomarker-guided treatment selection improves outcomes compared to treating all UHR individuals uniformly. Additionally, neuroimaging assessed before, during, and after preventive interventions could determine whether treatments that prevent conversion also normalize brain abnormalities, and whether biomarker changes mediate or predict treatment response—critical data for mechanism-based treatment development.

Sixth, extension to other severe mental disorders could test whether neuroimaging biomarkers predict not only psychotic but also affective and anxiety disorder onset in at-risk populations. Emerging evidence suggests that transdiagnostic neurobiological abnormalities cut across traditional diagnostic categories; neuroimaging assessed in individuals at clinical high risk for depression or bipolar disorder might identify shared versus disorder-specific brain signatures, potentially enabling precise prediction across the mental disorder spectrum rather than solely for psychosis. Critically, diagnostic specificity within psychosis conversions represents an important but neglected research direction. Only Fornito et al. [33] have formally tested differential prediction of the schizophrenia spectrum versus affective psychosis in UHR converters (*n* = 35:21 schizophrenia spectrum, 14 affective). Anterior cingulate cortex thinning predicted schizophrenia spectrum conversion (d = −0.79 to −0.91, *p* < 0.05) but not affective psychosis conversion, suggesting that prefrontal abnormalities may be relatively specific to schizophrenia spectrum risk. Most studies did not report diagnostic subtypes or had insufficient affective psychosis converters for differential analyses. Future research requires larger samples with both conversion types and preregistered hypotheses to identify diagnostically specific biomarker signatures, enabling personalized prevention strategies matched to likely diagnostic trajectories and optimal treatment approaches.

Finally, implementation science research examining barriers and facilitators to neuroimaging biomarker adoption in clinical settings would inform translation pathways. Key questions include: What level of predictive accuracy would clinicians and patients require to change management based on biomarker results? How should biomarker results be communicated to preserve hope while accurately conveying risk? What are the psychological impacts on individuals of receiving high-risk versus low-risk biomarker results? Can simplified imaging protocols or analysis pipelines reduce costs and increase accessibility? Could alternative non-invasive biomarkers (electroencephalography, retinal imaging, blood-based markers, digital phenotyping from smartphones) provide more scalable approaches for resource-limited settings?

### 4.6. Limitations

Several limitations qualify the conclusions of this systematic review. First, substantial clinical and methodological heterogeneity precluded quantitative meta-analysis, limiting the ability to provide pooled effect size estimates with confidence intervals. The narrative synthesis approach employed, while appropriate given the heterogeneity, relies more heavily on qualitative interpretation than quantitative meta-analysis. We acknowledge that with 25 included studies, quantitative meta-analysis using random-effects models would have been feasible for certain outcome measures, particularly for structural MRI findings in hippocampal and prefrontal regions, where sufficient studies reported comparable metrics. However, the substantial heterogeneity in analytical approaches (voxel-based morphometry vs. region-of-interest vs. surface-based methods), brain region definitions, and statistical thresholds across studies would have introduced considerable uncertainty into the pooled estimates. Future systematic reviews should prioritize meta-analysis where methodological standardization permits reliable quantitative synthesis. Additionally, when studies reported multiple comparable brain regions, we prioritized regions with the largest effect sizes for synthesis. While this approach highlights the most robust findings, it may overestimate overall effect magnitudes and should be considered when interpreting the strength of the evidence presented.

Second, we restricted inclusion to English-language publications, potentially missing relevant studies published in other languages, particularly from Asian, Eastern European, or Latin American research groups, though we note that major UHR research has been conducted primarily in English-speaking countries or international collaborations publishing in English.

Third, publication bias likely affects the literature, with positive findings more likely to be published than null results. We could not formally assess publication bias through funnel plots or statistical tests as these require meta-analytic pooling. The predominance of positive findings across the literature may overestimate true effect sizes if studies finding no neuroimaging differences between converters and non-converters were less likely to be published. However, several included studies [36,39,41,43] did not report conversion outcomes despite examining UHR populations, suggesting that not all null findings remained unpublished. Related to this argument is causal inference. While longitudinal designs establish the temporal precedence of brain alterations before psychosis onset, they cannot demonstrate causation. Alternative scenarios include the following: brain changes as consequences rather than causes of prodromal symptoms; shared factors independently causing both brain alterations and psychosis risk; reverse causation, whereby emerging psychotic experiences produce neuroplastic brain changes; and progressive changes as downstream rather than upstream effects. Establishing causality would require experimental interventions demonstrating that preventing brain alterations reduces conversion risk—evidence that does not exist

Fourth, nearly all included studies were conducted in high-income countries in Europe, North America, Australia, and East Asia, with minimal representation from low- and middle-income countries, Africa, or Latin America. Generalizability to populations differing in genetic background, environmental exposures, healthcare systems, and cultural factors remains uncertain. Given known neuroanatomical variation across populations related to genetic ancestry and that psychosis presentations show cultural variation, replication in diverse global populations is essential before widespread implementation.

Fifth, most studies examined relatively young UHR samples (mean ages 18–25 years), with limited data on older individuals meeting UHR criteria in middle adulthood, late-onset cases, or pediatric samples younger than 15 years. Age-related neurobiological changes during adolescent brain maturation may interact with psychosis-related pathology, and whether identified biomarkers generalize across the age spectrum requires investigation. Additionally, sex differences in neuroimaging findings were inconsistently examined. While most studies (92%) controlled for sex as a covariate, systematic extraction of sex-by-group interaction analyses was not performed in our review. This represents a limitation, as sex-specific effects in predicting conversion—given known sexual dimorphism in psychosis risk, age of onset, symptom profiles, and brain developmental trajectories—warrant dedicated systematic investigation.

Sixth, the focus on MRI-based biomarkers reflects the current research emphasis but may overlook other potentially valuable biomarkers including electroencephalography measures (event-related potentials, oscillatory power, connectivity), retinal imaging (given that the retina is embryologically derived from brain tissue and shows structural abnormalities in schizophrenia), peripheral biomarkers (inflammatory markers, oxidative stress indices, metabolomic signatures, microRNA profiles), or multimodal digital phenotyping from smartphones (speech patterns, physical activity, sleep–wake cycles, social interaction patterns). An exclusively neuroimaging focus may provide an incomplete picture of the full biomarker landscape.

Finally, our quality assessment using the Newcastle–Ottawa Scale adapted for neuroimaging studies, while systematic and conducted independently by two raters with high inter-rater reliability (κ = 0.92), involved some subjective judgments about study quality. Alternative quality assessment tools designed specifically for prognostic biomarker studies or neuroimaging studies might yield somewhat different quality ratings, though we believe our approach captured the most critical methodological features affecting the validity and reliability of the findings.

## 5. Conclusions

This systematic review synthesizing evidence from 25 longitudinal neuroimaging studies demonstrates that ultra-high risk individuals who subsequently develop psychosis exhibit detectable neurobiological abnormalities at baseline assessment, including medial temporal and prefrontal cortical volume reductions (d = −0.45 to −0.68), thalamocortical dysconnectivity (g = 0.66–0.88), white matter microstructural compromise, and elevated striatal glutamate (d = 0.76). Critically, converters experience dramatic progressive gray matter loss during the months immediately preceding conversion, establishing that active pathological processes operate during the prodromal-to-psychotic transition. Machine learning approaches integrating distributed neuroanatomical patterns across modalities achieve 80–90% classification accuracy, with multimodal models combining neuroimaging with clinical and cognitive measures achieving the highest predictive performance.

These findings establish neuroimaging biomarkers as among the most promising tools for precision prevention in early psychosis, enabling risk stratification, enrichment of prevention trials, mechanism-based treatment selection, and monitoring of treatment response. However, substantial work remains before clinical implementation, including larger multi-site validation studies, health economic analyses, development of simplified protocols and analysis pipelines, and randomized trials examining whether biomarker-guided treatment selection improves outcomes compared to standard care. The identified neurobiological signatures provide convergent support for dopaminergic, glutamatergic, and neuroinflammatory models of psychosis while also revealing the complexity and heterogeneity of the pathophysiological processes underlying psychosis emergence. Continued integration of neuroimaging with complementary biomarker modalities, mechanistic experimental medicine studies, and implementation science research will progressively transform neuroimaging from a research tool to a clinical instrument, enabling truly personalized approaches to psychosis prevention.

## Figures and Tables

**Table 1:** Characteristics of included studies.

Study	Country	N UHR (Conv)	Conv Rate	Follow-Up	Scanner	Modality	Quality
Pantelis 2003 [30]	Australia	75 (23)	30.7%	12 months	1.5T	sMRI	High (8/9)
Velakoulis 2006 [31]	Australia	51 (15)	29.4%	24–36 months	1.5T	sMRI	High (8/9)
Borgwardt 2007 [32]	Switzerland	35 (13)	37.1%	24 months	3T	sMRI	High (8/9)
Fornito 2008 [33]	Australia	70 (35)	50.0%	24 months	1.5T	sMRI	High (9/9)
Koutsouleris 2009 [34]	Germany	65 (21)	32.3%	48 months	1.5T	sMRI + ML	High (8/9)
Sun 2009 [35]	Australia	56 (21)	37.5%	12 months	1.5T	sMRI	High (8/9)
Stone 2009 [36]	UK	27 (N/R)	N/A	Cross-sect	3T	MRS	Moderate (6/9)
Takahashi 2009a [37]	Australia	97 (31)	32.0%	24 months	1.5T	sMRI	High (9/9)
Takahashi 2009b [38]	Australia	35 (12)	34.3%	Mean 1.8y	1.5T	sMRI	High (8/9)
Shim 2010 [39]	South Korea	19 (N/R)	N/A	Cross-sect	3T	rs-fMRI	Moderate (5/9)
Koutsouleris 2010 [40]	Germany	25 (N/R)	N/A	12 months	1.5T	sMRI + ML	High (7/9)
Lord 2011 [41]	UK	~30 (N/R)	N/A	Cross-sect	1.5T	task-fMRI	Moderate (6/9)
Mechelli 2011 [42]	UK	182 (35)	19.2%	24 months	1.5T	sMRI	High (9/9)
de la Fuente 2011 [43]	Mexico	18 (N/R)	N/A	Cross-sect	3T	MRS	High (7/9)
Allen 2016 [44]	UK	52 (~13)	~25%	17 months	3T	ASL/fMRI	High (8/9)
Carletti 2012 [45]	UK	32 (8)	25.0%	28 months	1.5T	DTI	High (8/9)
Dazzan 2012 [46]	UK	93 (28)	30.1%	24 months	3T	sMRI	High (8/9)
Koutsouleris 2012a [47]	Germany	48 (15)	31.3%	48 months	1.5T	Neurocog + ML	High (8/9)
Koutsouleris 2012b [48]	Switzerland	21 (16)	76.2%	48 months	3T	sMRI + ML	High (8/9)
Ziermans 2012 [49]	Netherlands	43 (12)	27.9%	12 months	1.5T	sMRI	High (8/9)
Takahashi 2014 [50]	Australia	135 (52)	38.5%	Variable	1.5T	sMRI	High (8/9)
Anticevic 2015 [51]	USA (8 sites)	243 (21)	8.6%	24 months	3T	rs-fMRI	High (9/9)
Koutsouleris 2015 [52]	Germany + Swiss	73 (~26)	~35%	54 months	1.5T/3T	sMRI + ML	High (8/9)
Cannon 2016 [53]	USA (8 sites)	274 (65)	23.7%	24 months	3T	sMRI + multi	High (9/9)
Chung 2018 [54]	South Korea	89 (24)	27.0%	24 months	3T	sMRI + MRS + ML	High (9/9)

**Table 2:** Structural MRI findings predicting psychosis conversion.

Study	Brain Region	Finding	Effect Size	Statistics
Pantelis 2003 [30]	Hippocampus (bilateral)	Reduced baseline volume in converters	d = −0.52 to −0.58	L: *p* = 0.01; R: *p* = 0.02
Pantelis 2003 [30]	Parahippocampal gyrus (L)	Progressive loss 4.8% in converters vs. 0.3% in non-converters	Large	*p* = 0.003
Velakoulis 2006 [31]	Hippocampus (L)	Reduced volume: 3.21 vs. 3.58 cm^3^	d = −0.56	*p* = 0.031
Velakoulis 2006 [31]	Parahippocampal gyrus (L)	Reduced volume: 2.84 vs. 3.18 cm^3^	d = −0.62	*p* = 0.019
Borgwardt 2007 [32]	Hippocampus (bilateral)	Baseline reduction + progressive loss over 24mo	d = −0.61 to −0.68	L: *p* = 0.008; R: *p* = 0.015
Mechelli 2011 [42]	Parahippocampal gyrus (R)	Reduced gray matter in converters (VBM)	d = −0.64	Z = 3.84, *p* < 0.001 FWE
Sun 2009 [35]	Inferior frontal gyrus (R)	Reduced baseline gray matter	d = −0.61	*p* = 0.004
Sun 2009 [35]	Middle frontal gyrus (bilateral)	Reduced baseline gray matter	d = −0.48 to −0.52	L: *p* = 0.018; R: *p* = 0.011
Fornito 2008 [33]	Anterior cingulate cortex	Bilateral rostral paralimbic ACC thinning	d = −0.68	HR = 2.84, *p* = 0.003
Dazzan 2012 [46]	Orbitofrontal cortex (L)	Reduced volume in converters	d = −0.52	*p* = 0.006; OR = 2.84
Mechelli 2011 [42]	Superior temporal gyrus (L)	Reduced gray matter in converters	d = −0.48	Z = 3.62, *p* = 0.003
Takahashi 2009b [38]	Superior temporal gyrus subregions	Progressive loss 2–6%/year in converters	Large	*p* < 0.05
Ziermans 2012 [49]	Superior temporal gyrus (R)	Progressive loss −2.8% vs. −0.6% annually	d = −0.72	*p* = 0.006
Takahashi 2009a [37]	Insula (bilateral)	Progressive atrophy −5.0%/year vs. −0.6%/year	Large	*p* < 0.001
Takahashi 2014 [50]	Olfactory sulcus	Shallower depth: 8.2 mm vs. 9.1 mm	d = −0.58	*p* = 0.003

**Table 3:** Functional MRI, DTI, and MRS findings predicting psychosis conversion.

Study	Modality	Brain Region/Network	Finding	Effect/Statistics
Allen 2016 [44]	task-fMRI	Prefrontal cortex, midbrain, hippocampus	Increased activation during verbal fluency	*p* < 0.05 FWE
Allen 2016 [44]	PET	Brainstem dopaminergic function	Elevated [18F]-DOPA uptake in converters	d = 0.82, *p* = 0.03
Shim 2010 [39]	rs-fMRI	Default mode network	DMN hyperconnectivity in UHR vs. controls	*p* < 0.05 FWE
Lord 2011 [41]	task-fMRI	Anterior cingulate cortex	Reduced ACC topological centrality in high-symptom ARMS	*p* < 0.05 FWE
Anticevic 2015 [51]	rs-fMRI	Thalamus-prefrontal connectivity	Hypoconnectivity with PFC/cerebellum in converters	g = 0.88, *p* < 0.001
Anticevic 2015 [51]	rs-fMRI	Thalamus-sensorimotor connectivity	Hyperconnectivity with sensorimotor cortex in converters	g = 0.66, *p* < 0.001
Carletti 2012 [45]	DTI	Left frontal white matter	Progressive FA reduction in converters	Group × time *p* < 0.001
Stone 2009 [36]	1H-MRS	Thalamus	Lower glutamate in ARMS vs. controls	*p* < 0.05; r > 0.5
Stone 2009 [36]	1H-MRS	Anterior cingulate cortex	Higher glutamine in ARMS vs. controls	*p* < 0.05
de la Fuente 2011 [43]	1H-MRS	Dorsal caudate (associative striatum)	Elevated glutamate in UHR vs. controls	d = 0.76, *p* = 0.003

Abbreviations: task-fMRI = task-based functional MRI; rs-fMRI = resting-state functional MRI; DTI = diffusion tensor imaging; 1H-MRS = proton magnetic resonance spectroscopy; PET = positron emission tomography; DMN = default mode network; ACC = anterior cingulate cortex; ARMS = at-risk mental state; PFC = prefrontal cortex; FA = fractional anisotropy; FWE = family-wise error correction; d = Cohen’s d; g = Hedges’ g.

## Data Availability

The original contributions presented in this study are included in the article. Further inquiries can be directed to the corresponding author.

## Supplementary Materials

The following supporting information can be downloaded at: https://www.mdpi.com/article/10.3390/brainsci16010112/s1, Table S1: Newcastle-Ottawa Scale Quality Assessment for Included Studies.

## Appendix A. Prisma 2020 Checklist

**Table A1:** 2020 reporting guidelines (Page et al.) [9] The systematic review protocol was prospectively registered with PROSPERO prior to initiating the systematic literature search.

Section/Topic	Item	Location Reported
**Title**	Identify the report as a systematic review	Title page
**Abstract**	Provide a structured summary including background, objectives, methods, results, and conclusions	Abstract
**Introduction—Rationale**	Describe the rationale for the review in the context of existing knowledge	Section 1, paragraphs 1–8
**Introduction—Objectives**	Provide explicit statement of objective(s) or question(s) the review addresses	Section 1, final paragraph
**Methods—Eligibility criteria**	Specify inclusion and exclusion criteria for the review including PICOS elements	Section 2, Section 2.2
**Methods—Information sources**	Specify all databases, registers, websites, and other sources searched	Section 2, Section 2.3; Appendix B
**Methods—Search strategy**	Present full search strategies for all databases and registers	Section 2, Section 2.3; Appendix B
**Methods—Selection process**	State the process for selecting studies including screening, eligibility, and inclusion in the review	Section 2, Section 2.4
**Methods—Data collection**	Describe methods used to collect data from reports and data items collected	Section 2, Section 2.5; Appendix C
**Methods—Risk of bias**	Specify methods used to assess risk of bias in included studies	Section 2, Section 2.6
**Methods—Synthesis methods**	Describe the methods used to synthesize results and justify choices made	Section 2, Section 2.7
**Results—Study selection**	Describe the results of the search and selection process with PRISMA flow diagram	Section 3, Section 3.1; Appendix B
**Results—Study characteristics**	Cite each included study and present characteristics	Section 3, Section 3.1; Table 1
**Results—Risk of bias**	Present assessments of risk of bias for each included study	Section 3, Section 3.1; Supplementary Table S1
**Results—Results of syntheses**	Present results of all statistical syntheses conducted	Section 3, Section 3.1 Table 2 and Table 3
**Discussion—Interpretation**	Provide interpretation of results in context of other evidence and implications	Section 4, Section 4.1, Section 4.2 and Section 4.3
**Discussion—Limitations**	Discuss limitations at study and outcome level, and at review level	Section 4, Section 4.4 and Section 4.6
**Discussion—Conclusions**	Provide general interpretation in the context of other evidence	Section 4, Section 4.6
**Other—Registration**	Provide registration information including register name and registration number	Section 2, Section 2.1
**Other—Funding**	Describe sources of financial or non-financial support for the review	Funding section

Inter-rater reliability: Cohen’s κ = 0.89 (95% CI: 0.84–0.94) for study selection, indicating excellent agreement between reviewers.

Note: All extracted data were entered into a standardized spreadsheet and double-checked for accuracy. Any discrepancies between reviewers were discussed and resolved by re-examining the original source publication. Study authors were contacted when data were missing or unclear, with two reminder emails sent at two-week intervals if no response was received to the initial contact.

## Appendix B. Database Search Strategies

**Database**	**Search Strategy**
**PubMed/MEDLINE** Date: 15 February 2025 Range: 2000–2025 **Results: 1247**	**#1 Population terms:** (“ultra high risk”[Title/Abstract] OR “ultra-high risk”[Title/Abstract] OR “clinical high risk”[Title/Abstract] OR “at risk mental state”[Title/Abstract] OR “at-risk mental state”[Title/Abstract] OR “prodromal psychosis”[Title/Abstract] OR “prodrome”[Title/Abstract] OR UHR[Title/Abstract] OR CHR[Title/Abstract] OR ARMS[Title/Abstract]) **#2 Neuroimaging terms:** (“magnetic resonance imaging”[MeSH Terms] OR MRI[Title/Abstract] OR “functional MRI”[Title/Abstract] OR fMRI[Title/Abstract] OR “diffusion tensor imaging”[Title/Abstract] OR DTI[Title/Abstract] OR “structural imaging”[Title/Abstract] OR “voxel-based morphometry”[Title/Abstract] OR VBM[Title/Abstract] OR “functional connectivity”[Title/Abstract] OR “resting state”[Title/Abstract] OR “resting-state”[Title/Abstract] OR “brain volume”[Title/Abstract] OR “cortical thickness”[Title/Abstract] OR “gray matter”[Title/Abstract] OR “grey matter”[Title/Abstract] OR “white matter”[Title/Abstract] OR “magnetic resonance spectroscopy”[Title/Abstract] OR MRS[Title/Abstract] OR neuroimaging[Title/Abstract]) **#3 Outcome terms:** (conversion[Title/Abstract] OR transition[Title/Abstract] OR “psychosis onset”[Title/Abstract] OR progression[Title/Abstract] OR “develop psychosis”[Title/Abstract] OR “convert to psychosis”[Title/Abstract] OR predict[Title/Abstract] OR biomarker[Title/Abstract]) **#4 Combined: #1 AND #2 AND #3** Filters: English language; 2000–2025; Journal Article
**Scopus** Date: 15 February 2025 Range: 2000–2025 **Results: 1456**	TITLE-ABS-KEY ((“ultra high risk” OR “ultra-high risk” OR “clinical high risk” OR “at risk mental state” OR “prodromal psychosis” OR uhr OR chr OR arms) AND (mri OR fmri OR “diffusion tensor” OR dti OR “structural imaging” OR “voxel-based morphometry” OR “functional connectivity” OR “resting state” OR “brain volume” OR “cortical thickness” OR “gray matter” OR “grey matter” OR “white matter” OR spectroscopy OR mrs OR neuroimaging) AND (conversion OR transition OR “psychosis onset” OR progression OR predict OR biomarker) **Limiters:** PUBYEAR > 1999 AND PUBYEAR < 2026 LIMIT-TO (LANGUAGE, “English”)
**Web of Science** Date: 15 February 2025 Range: 2000–2025 **Results: 982**	TS = ((“ultra high risk” OR “ultra-high risk” OR “clinical high risk” OR “at risk mental state” OR “prodromal psychosis” OR UHR OR CHR OR ARMS) AND (MRI OR fMRI OR “diffusion tensor” OR DTI OR “structural imaging” OR “functional connectivity” OR “resting state” OR “brain volume” OR “cortical thickness” OR “gray matter” OR “grey matter” OR “white matter” OR spectroscopy OR MRS OR neuroimaging) AND (conversion OR transition OR “psychosis onset” OR progression OR predict* OR biomarker) **Limiters:** Timespan: 2000–2025 Language: English Document types: Article
**PsycINFO** Date: 15 February 2025 Range: 2000–2025 **Results: 324**	AB ((“ultra high risk” OR “ultra-high risk” OR “clinical high risk” OR “at risk mental state” OR “prodromal psychosis” OR UHR OR CHR OR ARMS) AND (MRI OR fMRI OR “diffusion tensor” OR DTI OR “structural imaging” OR “functional connectivity” OR “resting state” OR “brain volume” OR “cortical thickness” OR “gray matter” OR “white matter” OR spectroscopy OR MRS OR neuroimaging) AND (conversion OR transition OR “psychosis onset” OR progression OR predict OR biomarker) **Limiters:** Published Date: 20000101–20250215 Language: English Document Type: Journal Article
**Cochrane Library** Date: 15 February 2025 Range: 2000–2025 **Results: 118**	(“ultra high risk” OR “clinical high risk” OR “at risk mental state” OR “prodromal psychosis” OR UHR OR CHR OR ARMS):ti, ab, kw **AND** (MRI OR fMRI OR “diffusion tensor” OR DTI OR neuroimaging OR “brain imaging” OR “structural imaging” OR “functional connectivity”):ti, ab, kw **AND** (conversion OR transition OR “psychosis onset” OR predict OR biomarker):ti, ab, kw
**TOTAL**	**Total Records from Database Searches: 4127** **Additional Records (Reference Lists + Citation Tracking): 23** **GRAND TOTAL: 4150 records**
	Inter-rater reliability: Cohen’s κ = 0.89 (95% CI: 0.84–0.94) for study selection, indicating excellent agreement between reviewers.

## Appendix C. Comprehensive Data Extraction Variables

**Category**	**Variables Extracted**
**Study Identification and Design**	- First author and publication year - Country and geographic location - Study design (prospective cohort, retrospective cohort) - Single-site versus multi-site study - Funding source and conflicts of interest
**Participant Characteristics**	- Total sample size (UHR participants) - Number of converters and non-converters - Conversion rate (percentage) - Age: mean, standard deviation, and range - Sex distribution (percentage male/female) - Ethnicity and racial composition - UHR criteria used (CAARMS, SIPS/SOPS, Basel, other) - UHR subtype distribution (APS, BLIPS, GRD) - Baseline symptom severity (SIPS, PANSS, CAARMS scores) - Baseline functioning (GAF, SOFAS scores) - Antipsychotic medication status at baseline (%, dosage) - Cannabis use (% users, frequency, lifetime exposure) - Other substance use - Psychiatric comorbidities (depression, anxiety, PTSD) - Family history of psychosis - Educational level
**Neuroimaging Assessment Details**	- Scanner manufacturer and model - Magnetic field strength (1.5T, 3T, other) - Imaging modality (sMRI, fMRI, DTI, MRS, multimodal) - Structural MRI sequence parameters (TR, TE, voxel size, slice thickness) - Functional MRI paradigm (task-based: specific task; resting-state: duration) - DTI sequence parameters (number of directions, b-values) - MRS voxel location, size, and metabolites measured - Brain regions assessed (specific regions or whole-brain) - Analysis approach (ROI, VBM, surface-based, tract-based, connectivity) - Software used for image processing and analysis - Quality control procedures - Head motion assessment and correction methods - Artifact detection and exclusion criteria
**Follow-up and Outcome Assessment**	- Follow-up duration (months or years) - Conversion assessment method and criteria (DSM-IV/5, ICD-10/11) - Assessment instruments used (SCID, CAARMS, SIPS) - Assessor training and inter-rater reliability - Blinding of assessors to neuroimaging results - Follow-up assessment schedule and frequency - Time to conversion for converters - Specific psychotic disorder diagnoses (schizophrenia, schizoaffective, brief psychotic, other) - Loss to follow-up (number and percentage) - Reasons for attrition
**Results and Effect Sizes**	- Neuroimaging measures: means and standard deviations for converters and non-converters - Statistical test results (*t*-tests, F-tests, Z-scores, *p*-values) - Effect sizes: Cohen’s d, Hedges’ g, partial eta-squared, other - Confidence intervals for effect sizes - Classification accuracy metrics: sensitivity, specificity - Positive predictive value (PPV) and negative predictive value (NPV) - Area under receiver operating characteristic curve (AUC-ROC) - Likelihood ratios (positive and negative) - Odds ratios and hazard ratios for conversion - Multivariable model results (beta coefficients, *p*-values) - Machine learning classification performance (accuracy, precision, recall, F1-score) - Cross-validation results - Feature importance or weights from machine learning models - Subgroup analyses (by sex, age, UHR subtype, medication status)
**Statistical Methods and Confounders**	- Statistical tests used - Multiple comparison correction methods (FWE, FDR, Bonferroni, other) - **Covariates** included **in analyses:** - Age correction - Sex as covariate - Total intracranial volume correction - Education level adjustment - Baseline symptom severity adjustment - Medication exposure correction - Cannabis use adjustment - Head motion parameters (for fMRI/DTI) - Handling of missing data - Power calculations and sample size justification - Statistical software used
	Note: All extracted data were entered into a standardized spreadsheet and double-checked for accuracy. Any discrepancies between reviewers were discussed and resolved by re-examining the original source publication. Study authors were contacted when data were missing or unclear, with two reminder emails sent at two-week intervals if no response was received to the initial contact.
