# Supplementary material for: Structural and Functional Neuroimaging Biomarkers as Predictors of Psychosis Conversion in Ultra-High Risk Individuals: A Systematic Review
Source: Brain Sci. 2026 Jan 20;16(1):112. doi: 10.3390/brainsci16010112 (PMC12838713; doi:10.3390/brainsci16010112)
Supplement: Supplementary file 1 [file brainsci-16-00112-s001.zip › brainsci-4083348-supplementary.pdf]

Supplementary Table S1: Newcastle-Ottawa Scale Quality Assessment for Included Studies

Scale: 0-9 stars. Quality: ≥7 stars = high quality (low risk of bias); 4-6 stars = moderate quality; <4 stars = low quality (high risk of bias).

SUMMARY: High quality (≥7 stars): 21 studies (84%); Moderate quality (4-6 stars): 4 studies (16%); Low quality (<4 stars): 0 studies (0%)

COMMON LIMITATIONS: Cross-sectional design (no conversion follow-up): 4 studies (16%); Limited comparability control: 4 studies (16%); Conversion outcomes not reported in some cross-sectional studies: 4 studies (16%); Blinding of outcome raters not explicitly stated: 15 studies (60%)

NOS CRITERIA: Selection (max 4 stars): Representativeness of UHR cohort, ascertainment using validated instruments, conversion not present at baseline, baseline group comparability; Comparability (max 2 stars): Control for confounders (age, sex, medication); Outcome (max 3 stars): Validated conversion assessment, adequate follow-up (≥12 months), completeness of follow-up

Study	Design	Selection /4	Comparability /2	Outcome /3	Total /9	Quality
**Pantelis 2003 [30]**	Prospective cohort	4	2	2	8	High quality
**Velakoulis 2006 [33]**	Prospective cohort	4	2	2	8	High quality
**Borgwardt 2007[32]**	Prospective cohort	4	2	2	8	High quality
**Fornito 2008 [38]**	Prospective cohort	4	2	3	9	High quality
**Koutsouleris 2009[478]**	Prospective cohort	4	2	2	8	High quality
**Sun 2009 [37]**	Prospective cohort	4	2	2	8	High quality
**Stone 2009 [46]**	Cross-sectional	3	1	2	6	Moderate quality
**Takahashi 2009a [34]**	Prospective cohort	4	2	3	9	High quality
**Takahashi 2009b [35]**	Prospective cohort	4	2	2	8	High quality
**Shim 2010 [42]**	Cross-sectional	3	1	1	5	Moderate quality
**Koutsouleris 2010 [49]**	Prospective cohort	4	2	1	7	High quality
**Lord 2011 [43]**	Cross-sectional	3	1	2	6	Moderate quality
**Mechelli 2011 [31]**	Prospective cohort	4	2	3	9	High quality
**de la Fuente 2011 [47]**	Cross-sectional	3	2	2	7	High quality
**Allen 2012 [41]**	Prospective cohort	4	2	2	8	High quality

**Carletti 2012 [44]**	Prospective cohort	4	2	2	8	High quality
**Dazzan 2012 [39]**	Prospective cohort	4	2	2	8	High quality
**Koutsouleris 2012a [50]**	Prospective cohort	4	2	2	8	High quality
**Koutsouleris 2012b[51]**	Retrospective cohort	4	2	2	8	High quality
**Ziermans 2012 [40]**	Prospective cohort	4	2	2	8	High quality
**Takahashi 2014 [34]**	Prospective cohort	4	2	2	8	High quality
**Anticevic 2015[44]**	Prospective cohort	4	2	3	9	High quality
**Koutsouleris 2015[52]**	Retrospective cohort	4	2	2	8	High quality
**Cannon 2016 [53]**	Prospective cohort	4	2	3	9	High quality
**Chung 2018 [54]**	Prospective cohort	4	2	3	9	High quality

NOTES: Inter-rater agreement for quality assessment: Cohen's κ = 0.92 (excellent agreement); Cross-sectional studies (Stone 2009, Shim 2010, Lord 2011, de la Fuente 2011) received lower selection/outcome scores due to absence of longitudinal conversion follow-up; Comparability scores reflect control for core confounders (age, sex, medication). Neuroimaging-specific factors (ICV, head motion) assessed separately as supplementary quality indicators; All longitudinal studies used validated instruments for both UHR ascertainment (CAARMS, SIPS, Basel) and conversion assessment (SCID, CAARMS, SIPS); Koutsouleris 2010 received 1 star for outcome due to short follow-up and incomplete conversion data reporting; Complete adapted Newcastle-Ottawa Scale criteria provided in Supplementary Material S2

DETAILED SCORING BREAKDOWN:

Selection Domain (4 stars): Full 4 stars: 22 studies with representative samples, validated criteria, clear baseline assessment, comparable groups. 3 stars: 3 cross-sectional studies (Stone, Shim, Lord) lacking longitudinal comparability.

Comparability Domain (2 stars): 2 stars: 21 studies controlling for ≥2 core confounders. 1 star: 4 studies (Stone, Shim, Lord, Koutsouleris 2010) controlling for only 1-2 confounders.

Outcome Domain (3 stars): 3 stars: 6 studies (Fornito, Takahashi 2009a, Mechelli, Anticevic, Cannon, Chung) with validated assessment, adequate follow-up, excellent retention. 2 stars: 15 studies with validated assessment and adequate follow-up. 1 star: Cross-sectional studies without conversion follow-up.
